# Data quality and feasibility of the Experience Sampling Method across the spectrum of severe psychiatric disorders: a protocol for a systematic review and meta-analysis

**DOI:** 10.1186/s13643-018-0673-1

**Published:** 2018-01-18

**Authors:** Hugo Vachon, Aki Rintala, Wolfgang Viechtbauer, Inez Myin-Germeys

**Affiliations:** 1 Department of Neurosciences, Center for Contextual Psychiatry, KU Leuven, Kapucijnenvoer 33 blok I, 3000 Leuven, Belgium; 2 Department of Psychiatry and Neuropsychology, School for Mental Health and Neuroscience, Maastricht University, P.O. Box 616 (VIJV1), 6200 MD Maastricht, The Netherlands

**Keywords:** Experience Sampling Method, Ecological Momentary Assessment, Compliance, Feasibility, Methodology, Systematic review, Meta-analysis, Psychotic disorder, Major depressive disorder, Bipolar disorder

## Abstract

**Background:**

Due to a number of methodological advantages and theoretical considerations, more and more studies in clinical psychology research employ the Experience Sampling Method (ESM) as a data collection technique. Despite this growing interest, the absence of methodological guidelines related to the use of ESM has resulted in a large heterogeneity of designs while the potential effects of the design itself on the response behavior of the participants remain unknown. The objectives of this systematic review are to investigate the associations between the design characteristics and the data quality and feasibility of studies relying on ESM in severe psychiatric disorders.

**Methods:**

We will search for all published studies using ambulatory assessment with patients suffering from major depressive disorder, bipolar disorder, and psychotic disorder or individuals at high risk for these disorders. Electronic database searches will be performed in PubMed and Web of Science with no restriction on the publication date. Two reviewers will independently screen original studies in a title/abstract phase and a full-text phase based on the inclusion criteria. The information related to the design and sample characteristics, data quality, and feasibility will be extracted. We will provide results in terms of a descriptive synthesis, and when applicable, a meta-analysis of the findings will be conducted.

**Discussion:**

Our results will attempt to highlight how the feasibility and data quality of ambulatory assessment might be related to the methodological characteristics of the study designs in severe psychiatric disorders. We will discuss these associations in different subsamples if sufficient data are available and will examine limitations in the reporting of the methods of ambulatory studies in the current literature.

**Systematic review registration:**

The protocol for this systematic review was registered on PROSPERO (PROSPERO 2017:CRD42017060322) and is available in full on the University of York website (http://www.crd.york.ac.uk/PROSPERO/display_record.asp?ID=CRD42017060322).

**Electronic supplementary material:**

The online version of this article (10.1186/s13643-018-0673-1) contains supplementary material, which is available to authorized users.

## Background

Several reasons can explain the growing interest of clinical research in ambulatory assessment methods. Patients are generally poor in providing accurate, global, retrospective reflections of real-life experiences and behaviors, which is especially problematic for depressed patients who suffer from cognitive impairment [1] and for psychotic patients who also experience a loss of reality testing [2]. Therefore, the symptomatic dynamics in patients, which are essential for improving the understanding of severe psychiatric disorders [3–6], cannot be captured precisely through retrospective assessments [7]. Moreover, symptoms occur naturally in the context of daily life and as such, assessing patients in a controlled environment might neglect the influence of context on a patient’s state (e.g., [8, 9]). In this perspective, a growing body of research is now relying on ambulatory assessment methods such as the Experience Sampling Method (ESM, [10]) or Ecological Momentary Assessment (EMA, [11]). Based on repeated and momentary self-evaluations in the individual’s life context, this methodology allows capturing the evolution of affects, cognitions, or behaviors over time with a high ecological validity, as it limits potential artifacts from laboratory settings or retrospective recall [7, 12–14].

Despite the rapid increase of the use of momentary assessment methods in clinical psychology and psychiatric research, methodological challenges related to the use of such methods remain. In particular, as this method repeatedly prompts individuals to rate their current affective and cognitive state in the context of their daily life, it is plausible that the repetition of self-evaluations induces reactivity (e.g., increased burden, fatigue, or awareness of cognitive and affective state). A number of studies have reported reactive effects to the repeated assessments in several populations including healthy participants [15, 16] and in individuals suffering from chronic pain [17], substance use disorders [18–21], depression [22], and anxiety disorder or psychosis [19]. These reactive effects could involve a loss engagement in the participants, decreasing both the feasibility and the data quality in studies relying on this assessment method. Yet, the factors influencing this reactivity have not been clearly defined. In patients suffering from chronic pain, the systematic review of Morren et al. [23] reported several associations between reactivity and (i) methodological parameters related to the design of ESM studies such as the length of the ESM questionnaire or the use of a financial compensation and (ii) the characteristics of the sample such as the age of the participants. In this view, methodological parameters related to the design of ESM studies or to the characteristics of the sampled population are likely to undermine the feasibility and the quality of the data collected. However, the systematic review of Morren et al. [23] focused on momentary assessments related to chronic pain and these observations are likely to differ when targeting different clinical populations. Particularly, this important topic has not been investigated in severe psychiatric disorders while numerous ESM-based studies have been performed in these clinical populations. Considering the heterogeneity of designs in the related literature (e.g., with respect to the duration of the follow-up, the amount of self-evaluations, or the type of device used for data collection), there is a crucial need to answer these methodological concerns.

Our objective is to investigate protocol- and subject-related factors that could undermine the feasibility of ambulatory studies and the quality of the data collected through this methodological framework in psychotic disorder (PD), major depressive disorder (MDD), bipolar disorder (BD), and high-risk individuals for these disorders (HR).

## Methods

This protocol is based on the PRISMA-P (Preferred Reporting Items for Systematic Review and Meta-Analysis Protocols) guidelines [24]. For transparency and completeness, a completed PRISMA-P 2015 checklist is provided as an additional file [see Additional file 1]. The protocol has also been registered in the International Prospective Register of Systematic Reviews database (PROSPERO 2017:CRD42017060322).

### Search strategy

A systematic literature search will be performed without publication time limit in PubMed and Web of Science. Observational as well as randomized controlled studies will be included. Case studies, case reports, protocols, study designs, and systematic reviews will not be considered. The search strategy was designed to include relevant terms regarding ambulatory and momentary assessments (e.g., “experience sampling method” and “ecological momentary assessment”) as well as terms related to the clinical diagnosis of the participants under study (e.g., “psychotic disorder”, “major depressive disorder”, “bipolar disorder”). The search strategy will use either MeSH or keyword heading. A concept plan was built with the identified keywords and descriptors to run the search [see Additional file 2]. Unpublished studies as well as studies published in another language than English will not be considered.

Only studies using ESM/EMA designs in PD, MDD, BD, and HR will be included in this systematic review. If available within the included studies, data from non-psychopathological/healthy control groups (HC) will be considered and will serve as a reference group. Ambulatory studies involving only a single daily assessment will be excluded as this time sampling is not representative of the repeated momentary assessment that defines ESM research. To determine the eligibility of the original studies, two researchers (HV and AR) will independently conduct the screening of the studies in the title/abstract and full-text phases based on the inclusion and exclusion criteria. The screening results will be compared in order to identify any discrepancies. In case of a disagreement, a third researcher (IMG) will be consulted. We will use the PRISMA flow diagram to report quantitative and qualitative information on every phase of the selection process.

### Data extraction

When available, data will be extracted for the following items: (i) general study characteristics (i.e., authors, title, year, study design); (ii) sample characteristics (i.e., number of participants included in the analysis, age, gender, clinical status, ethnicity, educational status, employment status, marital status, cohabiting status, medication); (iii) ambulatory design characteristics (i.e., number of momentary assessments per day, number of ambulatory assessment days, number of ambulatory assessment periods [continuous or intermittent assessment], delay between ambulatory assessment periods, ambulatory sampling method [fixed, semi-random or random sampling], time intervals between the ambulatory assessments within a day, time intervals between the first and the last ambulatory assessment within a day, time of the start and the end of the ambulatory assessments within a day, number of items in the ambulatory questionnaire, approximate mean duration of the ambulatory questionnaire, type of scales used in the ambulatory questionnaire, type of device used to perform the ambulatory assessment, type of incentive, amount of the incentive); and (iv) the attrition rate (proportion of individuals excluded from the study compared to the number of individuals included at baseline) and the compliance rate (proportion of self-evaluations completed by the participants compared to the theoretical maximal number of self-evaluations allowed by the design) of the included studies. When needed, the corresponding authors of the original studies will be contacted for further information. Data from the included studies will be extracted and stored in a customized spreadsheet structured according to the items mentioned above and that will be provided as part of the supplementary materials in the upcoming systematic review.

### Assessment of methodological quality and risk of bias

The current systematic review does not investigate the main outcomes of the studies that will be included. As such, the conventional assessment of methodological quality is not directly applicable. Instead, the quality of the reporting of the attrition and compliance rates in the included studies will be assessed independently by HV and AR. The scale and the scoring developed to perform this assessment will be derived from the four-point item of the Grades of Recommendation, Assessment, Development, and Evaluation Working Group [25] to fit the aims of this review. This scale will be defined clearly and provided as supplementary material in the upcoming systematic review. If needed, the corresponding authors of the original studies will be contacted for further information.

### Data analysis

Studies providing sufficient data regarding the main outcomes (i.e., attrition rates and compliance rates) will be analyzed. If data are not amendable to a statistical analysis, a qualitative synthesis of the main outcomes under study will be conducted. If a sufficient amount of commensurable data is available, a quantitative synthesis (meta-analysis) will be performed.

For attrition, we will analyze the reported/calculated proportions. For compliance, there is in principle a proportion (of completed self-evaluations) per participant, but this information is unlikely to be available. Instead, we will analyze the mean proportions (i.e., ${\overline{p}}_i=\left(\sum \limits_{j=1}^{n_i}{p}_{ij}\right)/{n}_i,$ where *p*_*ij*_ denotes the proportion of completed evaluations for the *j*th participant in the *i*th study and *n*_*i*_ the group size). We expect either ${\overline{p}}_i$ to be reported directly (either in terms of a proportion or percentage) or the total number of self-evaluations collected, which is easily converted to ${\overline{p}}_i$ (i.e., if ${\overline{p}}_i={x}_i/\left({n}_i\times {m}_i\right)$, where *x*_*i*_ denotes the total number of self-evaluations collected and *m*_*i*_ the theoretical maximal number of self-evaluations allowed by the design).

Both outcomes will be analyzed using a multilevel random-effects model [26] with random effects/intercepts for studies and groups within studies. Also, we will include the group type (factor with five levels: PD, MDD, BD, HR, HC) and differences in protocols as fixed effects in the model. The a priori planned analyses consist of (1) a comparison of groups with different clinical diagnoses (PD vs. MDD vs. BD vs. HR vs. HC), (2) a comparison of population groups (i.e., based on socio-demographic factors), and (3) the influence of protocol characteristics on the outcomes (e.g., low- vs. high-frequency ambulatory sampling in PD). Results will be reported in terms of 95% confidence intervals and (two-sided) *p* values for each outcome. Heterogeneity will be assessed using the *Q* test and the *I*-squared statistic. A qualitative synthesis summarizing the quality of the report of the main outcomes in the database will be provided.

## Discussion and conclusion

To date, little to no information is available regarding the methodological factors that could undermine the data quality and feasibility of ambulatory studies related to severe psychiatric disorders despite a large body of research relying on heterogeneous protocols. Assessing this question within an individual study would involve large samples following numerous protocols with varying parameters and as such would be particularly challenging. Accordingly, there is a need to conduct a meta-analysis to provide an exhaustive assessment of the available evidence using clear and reproducible methods. The objective of this systematic review is to synthesize knowledge surrounding the data quality of ambulatory designs in severe psychiatric disorders with respect to various methodological parameters and as a function of the participants’ clinical status. This systematic review aims to provide evidence-based information to researchers and clinicians on the design of ambulatory protocols and, if applicable, to propose guidelines in order to improve the quality of the data collected in this context.

## Additional files

Additional file 1: PRISMA-P (Preferred Reporting Items for Systematic review and Meta-Analysis Protocols) 2015 checklist: recommended items to address in a systematic review protocol. (DOC 55 kb)

Additional file 2: Concept plan of the search strategy. (DOCX 12 kb)

## Abbreviations

BD: Bipolar disorder
ESM: Experience Sampling Method
HC: Healthy controls
HR: High-risk for severe psychiatric disorders
MDD: Major depressive disorder
PD: Psychotic disorder
PRISMA: Preferred Reporting Items for Systematic Reviews and Meta-Analyses
PROSPERO: International Prospective Register of Systematic Reviews
