# Supplementary material for: Data quality and feasibility of the Experience Sampling Method across the spectrum of severe psychiatric disorders: a protocol for a systematic review and meta-analysis
Source: Syst Rev. 2018 Jan 18;7:7. doi: 10.1186/s13643-018-0673-1 (PMC5774093; doi:10.1186/s13643-018-0673-1)
Supplement: Supplementary file 2 — Concept plan of the search strategy. (DOCX 12 kb) [file 13643_2018_673_MOESM2_ESM.docx]

**Additional file 2**

Concept plan of the search strategy

| N0 | Query |
| --- | --- |
| 1 | (Major depressive disorder) AND (((((Momentary assessment) OR Experience sampling method) OR Ecological momentary assessment) OR Ambulatory study) OR Diary study) |
| 2 | (Depression) AND (((((Momentary assessment) OR Experience sampling method) OR Ecological momentary assessment) OR Ambulatory study) OR Diary study) |
| 3 | (Bipolar disorder) AND (((((Momentary assessment) OR Experience sampling method) OR Ecological momentary assessment) OR Ambulatory study) OR Diary study) |
| 4 | (Psychotic disorder) AND (((((Momentary assessment) OR Experience sampling method) OR Ecological momentary assessment) OR Ambulatory study) OR Diary study) |
| 5 | (Psychosis) AND (((((Momentary assessment) OR Experience sampling method) OR Ecological momentary assessment) OR Ambulatory study) OR Diary study) |
| 6 | (Schizophrenia) AND (((((Momentary assessment) OR Experience sampling method) OR Ecological momentary assessment) OR Ambulatory study) OR Diary study) |
